# What could keep young people away from alcohol and cigarettes? Findings from the UK Household Longitudinal Study

**DOI:** 10.1186/s12889-017-4284-x

**Published:** 2017-05-25

**Authors:** Noriko Cable, Maria Francisca Roman Mella, Yvonne Kelly

**Affiliations:** Department of Epidemiology and Public Health, University College London 1-19 Torrington Place, WC1E 6BT, London, UK

**Keywords:** Adolescent behaviour, Happiness, Harm awareness, UK Household Longitudinal Study

## Abstract

**Background:**

Adolescents are vulnerable to risky behaviours that are likely to co-occur. We examined whether happiness, awareness of alcohol- or smoking-related harm or the size of friendship networks would be longitudinally associated with young people’s risky behaviours.

**Methods:**

We used available cases (*N=*1,729) from adolescents aged between 10 and 15 who participated in waves 2 and 3 of the UK Longitudinal Household Study that has annually collected population representative data from 40,000 UK households. The outcome variable was patterns of cigarette and alcohol use among adolescents (1= persistent non-use; 2= ex-use; 3= initiation; 4= persistent use) that we derived by tabulating current alcohol or cigarette use at waves 2 and 3. Explanatory variables were scores on participants’ perception of overall happiness, awareness of harm due to alcohol and cigarette use, and supportive friendship network size, collected at wave 2. Covariates were participants’ sex, age, base level of self-reported health status, reported religious affiliation, and household social position. All estimates were corrected for the complex survey design and non-response. Multinomial logistic regression was used to test assumed associations by taking persistent cigarette and alcohol use as the reference category.

**Results:**

Findings showed higher happiness scores were longitudinally associated with adolescents’ persistent non-use (RRR=1.06, 95% CI=1.01-1.13). Awareness of alcohol or cigarette use-related harm was longitudinally associated with persistent non-use (RRR=1.24, 95% CI 1.15-1.35) as well as initiation of alcohol or cigarette use (RRR=1.21, 95% CI=1.11-1.32).

**Conclusion:**

Joint interventions to promote happiness and harm awareness could help young adolescents from engaging with drinking alcohol or smoking cigarettes.

## Background

Adolescence is a developmental stage, known to be vulnerable to engaging with risky health behaviours, including drinking alcohol [1] or smoking cigarettes [2]. A systematic review on alcohol harm showed a decreasing trend in alcohol consumption among adolescents in the UK [3]; a similar trend was also observed in cigarette use among school-aged children in England [4]. However, the profile of alcohol and cigarette use among older children showed a different picture, with a higher prevalence of alcohol and cigarette use [4] and an increased likelihood of physical harm occasioned by their risky alcohol use [3], compared to younger children. Drinking and smoking behaviours were likely to co-occur in the early stage of adolescence and to persist [5], increasing the risk of heavier alcohol consumption [6]. These findings warrants our attention in investigating potentially preventive factors for adolescent alcohol and cigarettes use.

### Awareness of alcohol- and cigarette-related harm

Recent work using data from the UK Millennium Cohort Study showed that awareness of harms due to alcohol was associated with a reduced risk of drinking among 11 year olds [7, 8]. School policies that provide knowledge about the harms of cigarette use were effective in altering school children’s perceptions [9]. Moreover, school-based interventions on alcohol education and counselling were found to be effective in preventing school-aged children from engaging in risky alcohol use [10]. Therefore, estimating the potentially protective effect of harm awareness on alcohol or cigarette use in a contemporary UK setting could help inform harm reduction strategies.

### Other possible protective factors

Recent findings suggest potential influences for adolescents’ psychosocial factors in relation to their alcohol or cigarette use. Hostility [11] and emotional distress [12] were identified characteristics of school-aged (between 10 and 19) smokers, while emotional distress preceded or was concurrent with the patterns of alcohol and tobacco use among adolescents, aged between 15 and 18 [13]. Social isolation was another identified characteristic linking to adolescent cigarette use [14].

Booker and others [12] showed that self-reported feelings of happiness had an independent, cross-sectional association with a range of health-related behaviours such as drinking alcohol, smoking cigarettes, exercise, and diet. However, prospective findings are sparse here and this link needs to be examined further to establish a temporal order by examining whether happiness could protect children from using alcohol or cigarettes. Religiosity has been linked with adolescents’ health outcomes [15]. Findings linking between religiosity and substance use were inconsistent, given the multidimensional nature of the concept [16]. Adolescents’ religious affiliation was linked with alcohol use [16] and had shown to be predictive of their lifestyle [17]. These findings suggest that adjustment of religious affiliation is required to examine the independent association between adolescents’ happiness and alcohol and cigarette use.

Despite peer drinking being a large driving factor for alcohol use among adolescents [1, 7, 8], it may be that jointly with happiness, larger supportive friendship networks could have the effect of distancing children from engaging with the use of alcohol or cigarettes. The aim of this study is to examine the roles of three possible protective factors (harm awareness, happiness, and supportive networks) in relation to patterns of alcohol or cigarette use among young people in the UK, based on a population-based household study. In this study, we specifically address the hypothesis, anticipating an independent longitudinal associations between these protective factors and non-initiation of alcohol and cigarette use among them.

## Methods

### Data

We used available cases (*N=*1,729) from young people between aged 10 and 15 who participated in both waves 2 and 3 [18] of Understanding Society (UK Longitudinal Household Study, UKHLS). UKHLS has collected data representative of the population from a selected 40,000 households in the United Kingdom annually since 2009 [19]. The study invites anybody in the household aged 16 and over to participate in the main survey. Separately, young people aged between 10 and 15 in the same household are asked to take part in the study by completing a short questionnaire. The field work was conducted during January 2010 and March 2012 for the wave 2 and during January 2011 and July 2013 for the wave 3. Further details regarding the sampling of UKHLS is found in the work by Burton et al. [19] Parental consent was obtained to collect data from eligible young people in the selected households [20]. Each child completed a paper and pencil questionnaire which was expected to take 10 minutes to complete, without parents being present [12]. Survey weights have been derived to correct estimates to reflect representativeness of the UK population, accounting for oversampling of minority groups and response patterns [21].

We were able to match 3,019 longitudinal cases between waves 2 (*n=*5,020) and 3 (*n=*4,427); Out of these cases, we excluded a further 1,290 cases that did not have information on any of the study variables; the final sample size for this study is 1729.

### Ethical consideration

UKHLS data are publicly available via the UK data archive (http://ukdataservice.ac.uk/) for academic use. All personal information that may link the data with the participants were removed already and therefore are exempt from obtaining ethical approval to use the data.

### Outcome

Alcohol and cigarette use status was approximated via participants’ response to their alcohol and cigarette use asked about in both waves 2 and 3. We coded the positive response to the use of either substance as ‘use’, as opposed to not using either of the substances (i.e. ‘non-use’). We combined responses at both waves and created four use categories as: persistent non-use (=not using alcohol or cigarettes at both waves), ex-use (=used alcohol or cigarettes at wave 2, but non-use at wave 3), initiation (=non-use at wave 2, but started using alcohol or cigarettes at wave 3), and persistent use (=used alcohol or cigarettes at both waves).

### Explanatory variables

#### Happiness

In UKHLS young participants’ degree of happiness was assessed in 6 areas of their lives: their school performance, looks, family, friends, school, and life in general. The participants were asked to identify their perceived happiness from 7 levels of smiling pictured face (i.e. emoticons), 1 being very happy and 7 being unhappy. We reversed each code for the high score to correspond with greater happiness. We adopted the approach of previous studies [12, 22] of treating these scales as an index by adding the sum of each scale. Cronbach’s alpha for this index was 0.75. It did not have substantial difference with age, ranging between 0.69 and 0.76 for ages 10 and 11, while the values for ages 12 to 15 fell within the range.

#### Harm awareness

We used questions concerning participants’ awareness about the harm caused by various types of alcohol or cigarette use that were administered at wave 2. They were asked to choose a response from no risk (=1) to great risk (=4) on occasional smoking, daily smoking, light daily drinking, heavy daily drinking, and heavy weekend drinking. Similarly to our approach towards the happiness variable, we applied principal component analysis to assess whether these items would be integrated under one dimension. The results of principal component analysis confirmed the unidimensionality of psychometric property on these items and we treated them as an index by summing up each item. Cronbach’s alpha of this index was 0.74 (range 0.61-0.81 for ages 14+ and 10 respectively).

#### Size of supportive friendship networks

At the wave 2 survey, participants were asked to identify the number of their friends with whom they could emotionally confide. The reported numbers ranged from 0 to 97 and were highly skewed from 7 onwards. In our study sample, nobody reported an absence of friends. We combined the upper tail of the distribution to create a 6+ category.

### Covariates

We included sex of the participants, age, religious affiliations, health status, and household social position in the model to account for those effects on the associations between the explanatory variables and outcome.

Past studies showed diminishing effects of parental influence on young people during the transitional developmental stage to enter adulthood [23, 24]. Our primary focus is to examine the roles of explanatory variables, and information about peer drinking is not available at the wave 2 survey. Therefore we did not include parental or peer alcohol use in the model to minimise added complexity to the model. Neither did we include participants’ residential characteristics (=urban or rural) because this factor was not significantly associated with the outcome in our preliminary analysis.

We used participants’ age at wave 2 in the model. Participants’ religious affiliations were only asked at wave 3. We treated this item as a binary response, 1 having religious affiliation and 0 being no religious affiliations. Health status of the participants was assessed by their response to a general health question, scored as 1 excellent to 5 poor. We coded household social position was derived by taking the highest occupational position of the parents that was reported by parents and indicated by the National Statistics Socio-economic classification (NS-SEC).

### Analyses

We used multinomial logistic regression to assess the presence of longitudinal associations between three explanatory variables (harm awareness, happiness, and size of friendship networks) and the outcome (use categories), taking persistent use as a reference category to address our study aim, especially the relationships between protective factors with the non-initiation of drinking and smoking.

Stata v13 SE [25] was used for the analysis. We used the ‘svy’ command to account for the complex data structure as well as applying the survey weights to the estimates. Variance was scaled to a single sampling unit to deal with the strata in the data by using the ‘single unit’ option in the ‘svy’ command.

## Results

Table 1 shows descriptive characteristics of the participants by their patterns of alcohol and cigarette use. Close to 70% (*n*=1190, weighted) of the participants were in the group of persistent non-use, while there were similar proportions (12.7%) in the groups of initiation (*n*=218.2, weighted) and persistent use (*n*=218, weighted). The group of ex-use was the smallest of all (*n*=92.91, weighted).

**Table 1:** Sample characteristics (weighted) presented separately by use statuses of the UKHLS youth participants (*N*=1,729)^a^

	Persistent non-use (*n*=1,190)	Ex-use (*n*=92.91)	Initiation (*n*=218.2)	Persistent use (*n*=218)
Mean (95%CI), Harm awareness	17.15 (16.95-17.34)	15.85 (15.04-16.66)	16.96 (16.62-17.31)	15.62 (15.19-16.05)
Mean (95%CI), Happiness	35.97 (35.57-36.37)	35.14 (34.28-35.99)	34.83 (34.11-35.56)	33.88 (32.89-34.87)
Friendship networks, %				
1	3.90	1.49	4.58	1.91
2	10.33	16.33	6.72	5.51
3	13.96	14.15	15.70	12.02
4	12.93	9.77	12.54	8.05
5	16.18	10.11	14.81	13.23
6+	42.70	48.13	45.65	59.26
Sex, %				
Boys	47.98	64.22	52.49	47.79
Girls	52.02	35.78	47.51	52.21
Age at base, %				
10	19.67	16.04	4.74	0.90
11	24.61	14.76	15.31	3.63
12	22.72	21.65	14.58	9.92
13	19.72	23.66	29.36	30.50
14+	13.27	23.89	36.00	55.05
Subjective health, very good or excellent %	71.21	68.53	62.76	55.83
Religious affiliations, present %	56.29	49.95	44.12	36.43
Managerial and professional occupational position, %	54.42	40.54	56.21	56.43

^a^Observed case, presented figures were based on the weighted count on each use status

As a whole, the number of participants was similar between boys (*n*=849.2, weighted) and girls (*n*= 869.6, weighted). Comparing the distribution of sex according to the use groups, more girls were in the groups of persistent use and non-use, while more boys were found in the groups of ex-use and initiation. Nevertheless, it was not statistically significant.

More young people aged between 10 and 12 were in the persistent non-use group, while participants aged 13 through 14 and over were in the persistent user and initiation groups, and these differences were statistically significant. Many of the young people, apart from those in the group of persistent use, rated their health as very good or excellent. Moreover, around half of the young people, apart from those in the group of ex-use, were from an affluent social background. However, the distribution of self-reported health or household position across use groups was not statistically significant.

In descriptive findings, young people in the persistent non-use group showed the highest mean score on harm awareness or happiness compared to the rest of the groups (Table 1). In regard to the size of friendship networks, most of the young people had more than 6 friends in whom they could emotionally confide. The results of multinomial logistic regression confirmed that adolescents’ happiness and harm awareness were differently associated with their use statuses (Table 2). In reference to persistent non-use, happiness was significantly and longitudinally associated with adolescents’ persistent non-use. After accounting for the effects from covariates and other explanatory variables, the association remained significant; one point increase in happiness score was equated with the relative risk ratio of 1.06 being in the group of persistent non-use relative to persistent use.

**Table 2:** Estimates (RRR) obtained from multinomial logistic regression with 95%CI showing the association between adolescents’ attitudes, happiness, and friend networks with use status among the USOC youth aged 10-15 (*N=*1,729)

	Persistent non-use			Ex-use			Initiation			Persistent use
	Model 1^a^	Model 2^b^	Model 3^c^	Model 1	Model 2	Model 3	Model 1	Model 2	Model 3	
Happiness	1.07 (1.02-1.14)	1.08 (1.02-1.14)	1.06 (1.01-1.13)	1.04 (0.97-1.10)	1.05 (0.98-1.11)	1.04 (0.97-1.10)	1.03 (0.98-1.09)	1.04 (0.98-1.10)	1.02 (0.96-1.08)	1.00
Harm awareness	1.26 (1.16-1.36)	1.25 (1.15-1.35)	1.24 (1.15-1.35)	1.07 (0.98-1.17)	1.07 (0.98-1.17)	1.07 (0.98-1.17)	1.21 (1.12-1.31)	1.21 (1.11-1.31)	1.21 (1.11-1.32)	1.00
Friendship networks	0.84 (0.69-1.02)	0.82 (0.67-1.01)	0.81 (0.67-1.01)	0.82 (0.66-1.02)	0.82 (0.66-1.02)	0.82 (0.65-1.02)	0.84 (0.69-1.02)	0.83 (0.68-1.02)	0.83 (0.68-1.02)	1.00

Note: ^a^sex and age; ^b^Model 1 + explanatory variables; ^c^ Model 2 + household occupational position, religious affliations, self-reported health at base

In addition, awareness of alcohol or cigarette use-related harm was positively and longitudinally associated with persistent non-use as well as with initiation. In the fully adjusted model, the relative risk ratios were similar between persistent non-use (RRR=1.24, 95% CI 1.14-1.35) and initiation (RRR=1.21, 95% CI=1.11-1.32) in reference to persistent use.

The size of friendship networks was not significantly associated with any of the use groups, and none of the explanatory variables were longitudinally associated with ex-use. However, Wald tests confirmed all of the outcome categories were significantly different from each other at the 0.01 level (Table 3), indicating that no outcome categories were to be combined.

**Table 3:** Wald test results, examining distinct differences across the outcome categories

	F	df	*p*-values
Persistent use vs. Persistent non-use	10.14	8	<0.001
Persistent use vs. Ex-use	5.60	8	<0.001
Persistent use vs. Initiation	3.74	8	<0.001
Persistent non-use vs. Ex-use	3.38	8	<0.001
Persistent non-use vs. Initiation	7.36	8	<0.001
Initiation vs. Ex-use	2.80	8	0.005

## Discussion

We found that adolescents’ positive affect, indicated by happiness and awareness of alcohol or cigarette use-related harm had longitudinal associations with persistent non-use as we hypothesised. Our finding offers extended support to the study by Booker et al. [12], reporting a cross-sectional association between these factors.

We also found that adolescents’ awareness of harm resulting from alcohol and cigarette use was significantly related to both initiation and persistent non-use. Prior work has shown that alcohol harm awareness is linked to a reduced chance of drinking in early adolescence [7, 8]. Adolescents’ expectations of gaining positive experiences with enhanced socialisation and reduced anxiety through alcohol use (i.e. positive expectancies) were found to be associated with their alcohol use [1, 7, 8]. Information about adolescents’ expectancies about alcohol or cigarettes was not collected in the UKHLS survey; however, it is possible that their higher expectations from drinking alcohol or smoking cigarettes may have overcome their awareness of harm from using those substances.

We expected to find protective associations between the size of adolescents’ supportive friendship networks and the patterns of their use. Huang et al. [26] found that the number of close friends obtained via social network sites was not associated with alcohol use among adolescents in the United States. However, due to data availability we do not have detailed information about whether the study participants’ friendship networks are ‘virtual’. Nor do we have data about peer drinking. Further studies exploring peer usage of alcohol and cigarettes within the friendship networks, and changes in friendship networks are needed to examine how adolescents’ alcohol and cigarette use takes place within the context of friendship networks.

## Strengths and limitations

In our study, we were able to statistically control covariates and all reported longitudinal associations were independent from participants’ sex, age, religious affiliation, health status, and household social position. As suggested by Rowland et al. [27], we considered the effect of the density of alcohol retail shops on the study’s outcome. In our preliminary analysis, geographical location of adolescents (urban or rural) was not significantly associated with the outcome. All estimates in our analyses took account of the complexity of the UKHLS sampling framework to reflect representativeness of the UK population.

We did not find that any explanatory variables were associated with stopping drinking or smoking. It is possible that recanting on alcohol or smoking may have affected the grouping of the outcome category even though smoking status obtained from male participants or those who started smoking at a young age has been shown elsewhere to be less prone to recall bias [28]. We only used data from waves 2 and 3 of the survey; using subsequent sweeps could have ascertained the validity of each status, especially ex-use. However, this would have led to reduced statistical power, due to attrition and exclusion of ineligible cases. In our study, we found statistical significance between all use groups, suggesting that each status is distinctively different from all the others.

Concerning the patterns of alcohol and cigarette use, attendance to church service or involvement in church activities (i.e. public religiosity) was found to be associated with adolescents’ cessation of cigarette use [29]. We only had information about participants’ religious affiliation, and it was treated as a covariate to ascertain independent associations between explanatory factors and patterns of adolescents’ alcohol and cigarette use. Social aspects of alcohol and cigarette use could have been explored if we had a theoretically validated measurement for religiosity.

In our study adolescents’ happiness and greater awareness of harm related to alcohol or cigarette use appeared to be jointly associated with their persistence in not engaging with drinking alcohol or smoking cigarettes. However, all information were collected via a self-reported questionnaire. Therefore significant findings should be interpreted with caution.

In UKHLS, adolescents who turn 16 at the data collection of each sweep are included in the main survey which contains detailed information about health-related behaviours; longitudinal analysis from early adolescence to entering into adulthood would look further at this longitudinal association as well as adding to our understanding of the formation and progression of alcohol and cigarette use in this population.

## Conclusions

In summary, adolescents’ feelings of happiness and harm awareness appear to be jointly important for their persistent non-engagement with drinking alcohol or smoking cigarettes. Preventive interventions for alcohol or cigarette use targeting this population could be aimed at promoting their level of well-being as well as equipping them with an awareness of alcohol- or smoking-related harm.

## Abbreviation

UKHLS: UK Household Longitudinal Study
